# Low-density SNP markers with high prediction accuracy of genomic selection for bacterial wilt resistance in tomato

**DOI:** 10.3389/fpls.2024.1402693

**Published:** 2024-05-30

**Authors:** Jeyun Yeon, Ngoc Thi Le, Jaehun Heo, Sung-Chur Sim

**Affiliations:** ^1^ Department of Bioindustry and Bioresource Engineering, Sejong University, Seoul, Republic of Korea; ^2^ Plant Engineering Research Institute, Sejong University, Seoul, Republic of Korea

**Keywords:** bacterial disease, prediction model, molecular marker, vegetable, breeding

## Abstract

Bacterial wilt (BW) is a soil-borne disease that leads to severe damage in tomato. Host resistance against BW is considered polygenic and effective in controlling this destructive disease. In this study, genomic selection (GS), which is a promising breeding strategy to improve quantitative traits, was investigated for BW resistance. Two tomato collections, TGC1 (*n* = 162) and TGC2 (*n* = 191), were used as training populations. Disease severity was assessed using three seedling assays in each population, and the best linear unbiased prediction (BLUP) values were obtained. The 31,142 SNP data were generated using the 51K Axiom array™ in the training populations. With these data, six GS models were trained to predict genomic estimated breeding values (GEBVs) in three populations (TGC1, TGC2, and combined). The parametric models Bayesian LASSO and RR-BLUP resulted in higher levels of prediction accuracy compared with all the non-parametric models (RKHS, SVM, and random forest) in two training populations. To identify low-density markers, two subsets of 1,557 SNPs were filtered based on marker effects (Bayesian LASSO) and variable importance values (random forest) in the combined population. An additional subset was generated using 1,357 SNPs from a genome-wide association study. These subsets showed prediction accuracies of 0.699 to 0.756 in Bayesian LASSO and 0.670 to 0.682 in random forest, which were higher relative to the 31,142 SNPs (0.625 and 0.614). Moreover, high prediction accuracies (0.743 and 0.702) were found with a common set of 135 SNPs derived from the three subsets. The resulting low-density SNPs will be useful to develop a cost-effective GS strategy for BW resistance in tomato breeding programs.

## 1 Introduction

Bacterial wilt (BW) is a soil-borne disease that is caused by *Ralstonia solanacearum* and leads to severe yield loss in major vegetables including tomato. After infection via roots, this pathogen rapidly colonizes the xylem of host plants, resulting in lethal wilting within several days (Vasse et al., 1995; Denny, 2000). *Ralstonia solanacearum* is distributed worldwide with five races, six biovars, and four phylotypes (Fegan and Prior, 2005; Denny, 2006). For cultivated tomato, the most virulent pathogens are race 1 (phylotypes I and II) and race 3 (phylotype II) in temperate regions (Carmeille et al., 2006). Chemical control is widely used to eliminate this pathogen but is often ineffective due to bacterial localization in deep soil (Sharma et al., 2021). As an alternative control strategy, using host resistance is cost-effective and environment-friendly. Through extensive studies in tomato and other crop species, BW resistance was found to have polygenic inheritance (Hayward, 1991).

Different levels of BW resistance were found in several tomato varieties including Hawaii 7996 (Ha7996), which is an important source of polygenic resistance (Danesh et al., 1994; Wang et al., 1998). These varieties have been used to investigate quantitative trait loci (QTL) in bi-parental mapping populations (Danesh et al., 1994; Thoquet et al., 1996a; Thoquet et al., 1996b; Wang et al., 2000; Carmeille et al., 2006; Wang et al., 2013; Truong et al., 2015). Consequently, several QTL associated with BW resistance have been found in previous studies. Of these, two loci on chromosomes 6 and 12, named *Bwr-6* and *Bwr-12*, were identified as major QTL for stable resistance against BW, explaining up to 22.2% and 56.1% of total phenotypic variations (Wang et al., 2013). In addition, a genome-wide association study (GWAS) in a collection of diverse tomato varieties revealed another major QTL on chromosome 4 along with *Bwr-6* and *Bwr-12* (Nguyen et al., 2021). These QTL studies also identified several loci with minor effects on different chromosomes that could be environment- or race-specific.

Major QTL have been used to improve BW resistance via marker-assisted selection (MAS) in tomato breeding programs. However, this approach has a limitation for minor QTL with small effects (Goddard, 2009; Phan and Sim, 2017). Genomic selection (GS) is considered an effective method to improve complex quantitative traits that are regulated by a large number of QTL (Meuwissen et al., 2001). For GS, genome-wide single nucleotide polymorphisms (SNPs) are used to predict genomic estimated breeding values (GEBVs) of breeding lines (Bernardo and Yu, 2007; Heffner et al., 2009; Crossa et al., 2010). Therefore, the prediction accuracy of GEBVs is a key to select breeding lines with favorable traits. To estimate GEBVs, parametric and non-parametric GS models have been developed, and their performances depend on the genetic architecture of traits (Zhong et al., 2009; Daetwyler et al., 2010; Merrick and Carter, 2021). Training population and marker density are other factors to determine the prediction accuracy of GEBVs (Heffner et al., 2009; Heffner et al., 2011a, Heffner et al., 2011b; Desta and Ortiz, 2014; Crossa et al., 2017). GEBVs with high levels of accuracy can be obtained using training populations consisting of individuals with diverse genetic backgrounds. Although large numbers of markers across genomes lead to high prediction accuracy, the effect of marker density can be variable for species and traits (Hao et al., 2019; Juliana et al., 2019).

For tomato, GS was investigated for fruit traits, metabolic traits, yield, earliness, heat tolerance, and bacterial spot resistance (Duangjit et al., 2016; Hernández-Bautista et al., 2016; Yamamoto et al., 2016; Yamamoto et al., 2017; Liabeuf et al., 2018; Hernández-Bautista et al., 2020; Cappetta et al., 2021; Tong et al., 2022). However, a cost-effective strategy with high prediction accuracy is still required to accelerate GS. In addition, BW resistance has been less studied for GS relative to other traits in tomato, even though MAS has been limited to major QTL. Therefore, the objective of this study was to assess the prediction accuracy of GEBVs for BW resistance and increase the efficiency of GS using low-density SNP markers in tomato. We used two tomato germplasm collections (TGC1 and TGC2) as training populations for phenotyping and genotyping. Each population was independently evaluated for BW resistance based on three seeding assays and was genotyped using the 51K SNP array. The TGC1 and TGC2 data were combined to generate a large training population, and the phenotypic data were adjusted using best linear unbiased prediction (BLUP). With these data, the prediction ability of six GS models was compared in the three training populations (TGC1, TGC2, and combined). Furthermore, the five subsets of markers were generated from the 31,142 SNPs using different methods and were evaluated for prediction accuracy using the selected models. All the subsets showed higher levels of prediction accuracy compared with the 31,142 SNPs, suggesting that low-density markers can be effective for GS. Our results will facilitate GS-based prediction of BW resistance in tomato breeding programs.

## 2 Materials and methods

### 2.1 Plant materials

Two tomato germplasm collections, TGC1 (*n* = 162) and TGC2 (*n* = 191), were used in this study. The TGC1 accessions included 119 determinate breeding lines, 42 semi-determinate breeding lines, and one undetermined breeding line from a private breeding program, originating from seven different countries (**Supplementary Table S1**). For TGC2 representing indeterminate accessions, 98 breeding lines were derived from the National Institute of Horticultural and Herbal Science (NIHHS) in Rural Development Administration (RDA), the Republic of Korea (ROK). The other 93 accessions were collected from the National Agrobiodiversity Center (NAC) in RDA, the Germplasm Resources Information Network (GRIN) in the U.S. Department of Agriculture, the C. M. Rick Tomato Genetics Resource Center (TGRC), and Sejong University (**Supplementary Table S1**).

### 2.2 Disease evaluation

These collections were independently evaluated for BW resistance at the seedling stage in a greenhouse. Three seedling assays for each collection were conducted with artificial inoculation of a virulent strain WR-1 (race 1, biovar 3, and phylotype I) in different seasons (spring, summer, and fall). For inoculum, this strain was cultured in the Difco™ nutrient broth medium containing 3 g/L of beef extract and 5 g/L of peptone (BD, Sparks, MD, USA) at 28°C for 48 h. Bacterial cells were collected and resuspended in sterile, double-distilled water, and the resulting suspension was standardized to OD_600 _= 0.3 (10^8^ CFU/mL) using the NanoDrop™ One spectrophotometer (Thermo Fisher Scientific, Waltham, MA, USA). The roots of 6- to 8-week-old seedlings were wounded by cutting for inoculation and then dipped in the bacterial suspension for 30 min (Carmeille et al., 2006). For each assay, five to seven seedlings per tomato accession were inoculated and then placed in the greenhouse with a randomized complete block design. Wilting symptoms were scored 10 or 14 days after inoculation using a 1–5 scale, where 1 = no wilting symptom, 2 = one or two leaves wilted, 3 = most of the leaves wilted, 4 = all the leaves wilted, and 5 = plant died (Kelman, 1953). To correct environmental effects between assays, the BLUP values were calculated for the phenotypic data of BW resistance using the R package “lme4” (Bates et al., 2015) and used for further analysis.

### 2.3 Genotyping

Genomic DNA of each accession was isolated from fresh and young leaf tissue of 3- to 4-week-old seedlings using a modified cetyl trimethyl ammonium bromide (CTAB) method (Kabelka et al., 2002). The isolated DNA pellets were resuspended by T_1/10_E buffer (10 mM of Tris–HCl pH 8.0, 0.1 mM of EDTA), and their concentrations were estimated using the NanoDrop™ One spectrophotometer (Thermo Fisher Scientific, Waltham, MA, USA). The final concentration was adjusted to 50 ng/µL for genotyping with the Axiom^®^ tomato array containing 51,912 SNPs (Yamamoto et al., 2016). For this SNP array-based genotyping, 200 ng of genomic DNA for each accession was amplified and then fragmented into 25–125 bp using the Axiom^®^ 2.0 reagent kit (Thermo Fisher Scientific, Waltham, MA, USA). The resulting DNA fragments were hybridized to the array in the Affymetrix^®^ GeneTitan system according to the manufacturer’s instructions. SNP calling was conducted using the Affymetirx^®^ Power Tools software package v1.18, and high-quality SNPs were filtered based on <10% of missing data and >5% of minor allele frequency. The remaining missing data of these SNPs were imputed using BEAGLE v5 with default parameter settings (Browning, 2008).

### 2.4 Assessment of GS model performance

Six GS models were used to predict GEBVs for BW resistance in three training populations (TGC1, TGC2, and combined). Of these, the parametric models included ridge regression-best linear unbiased prediction (RR-BLUP), BayesA, and Bayesian LASSO. The non-parametric models were reproducing kernel Hilbert space (RKHS), support vector machine (SVM), and random forest. The SNP genotypes were used to train the GS models along with the BLUP values for BW resistance in each population. The estimates of GEBVs were obtained in the GS models implemented in several R packages: “rrBLUP” for RR-BLUP and RKHS (Endelman, 2011), “BGLR” for BayesA and Bayesian LASSO (Pérez and De Los Campos, 2014), “e1071” for SVM (Meyer et al., 2023), and “randomForest” for random forest (Liaw and Wiener, 2002). Cross-validation was conducted using the leave-one-out cross-validation (LOOCV) method (Molinaro et al., 2005). Prediction accuracy was determined based on the Pearson correlation coefficients between GEBVs and observed phenotypes (BLUP values) in the training populations.

### 2.5 Prediction accuracy analysis of low-density markers

The prediction accuracy of low-density markers was investigated using the selected models, which showed the best performance in each of the two groups (parametric and non-parametric). For this analysis, several subsets were generated by filtering the total marker set of confident 31,142 SNPs. The first subset was obtained based on the marker effect values of 31,142 SNPs that were determined by estimating the effects of SNP allele substitution in a parametric model. With a non-parametric model, the second subset was generated based on variable importance (VIM) values that were determined as the percentage of increased mean squared error (MSE) after this marker was randomly permuted in a new sample (Nicodemus et al., 2010). The marker effect and VIM values were calculated in the combined population and 1,557 of 31,142 SNPs were selected as the top 5% of marker effects or VIM values. The third subset consisted of SNPs that were significantly associated with BW resistance. For this subset, a GWAS was conducted in the combined population. Population structure was inferred to determine the best K (number of clusters) using the STRUCTURE v.2.3.4 program (Pritchard et al., 2000). The 10 Ks (1–10) were tested in 10 independent simulations for each K with a burn-in of 20,000 iterations and a run length of 100,000 iterations. With the resulting log-likelihood values, the best K was found in the delta K method (Evanno et al., 2005), and the corresponding membership coefficients of tomato accessions were used as the Q matrix. In addition, a kinship matrix was generated using the VanRaden algorithm (Vanraden, 2008) for association analysis. Marker–trait association was detected using a multilocus mixed model (MLMM) implemented in the genomic association and prediction integrated tool (GAPIT) (Lipka et al., 2012). In this model, both Q and kinship matrices were used as covariates to reduce false-positive associations due to population structure and familial relatedness (Yu and Buckler, 2006). Significant associations were determined using two thresholds, *P <*0.05 and *P <*0.005. Two additional subsets (fourth and fifth) were also generated using the first to third subsets. For the fourth subset, all of the SNPs in the three subsets were combined, while the common SNPs were used for the fifth subset. These five subsets and a total marker set (31,142 SNPs) were used to estimate GEBVs for BW resistance, and their prediction accuracies were evaluated in the combined population.

## 3 Results

### 3.1 Phenotypic variation of BW resistance in the tomato collections

**Figure 1:**
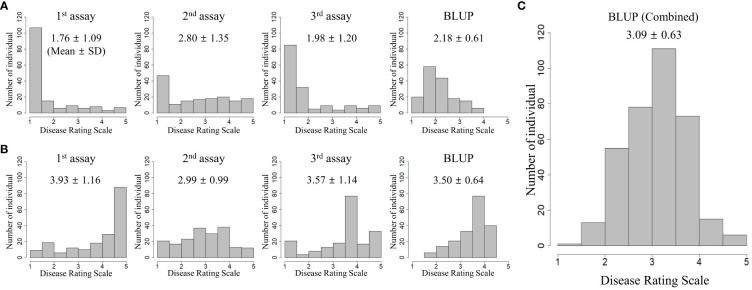
Phenotypic distributions of independent seedling assays and best linear unbiased prediction (BLUP) for bacterial wilt resistance in three populations: **(A)** TGC1 (*n* = 162), **(B)** TGC2 (*n* = 191), and **(C)** combined (*n* = 353). BLUP values were calculated by the random-effect model including genotype, location, year, and replication as variables to correct the environmental effects.

Two collections (TGC1 and TGC2) were evaluated for BW resistance in three independent greenhouse trials, respectively. For TGC1 (*n* = 162), we found asymmetric distributions skewed toward resistant responses in all the seedling assays (**Figure 1A**). The first and third assays, which were performed in spring and fall, showed the five rating scales (1 = no wilting symptom to 5 = plant died) with means of 1.76 and 1.98, respectively. The Pearson correlation coefficient was 0.69 between these assays (**Table 1**). The second assay conducted in summer showed a mean of 2.80 and relatively lower correlations with the first assay (0.16) and the third assay (0.36). The BLUP data of all the seedling assays showed less asymmetric distribution with a mean of 2.18 and correlation coefficients of 0.70 (vs. the second assay) to 0.87 (vs. the third assay) (**Figure 1A**, **Table 1**). For TGC2, the first and third assays showed skewed distributions to susceptible responses with means of 3.93 and 3.57, while the second assay showed a relatively symmetric distribution with a mean of 2.99 (**Figure 1B**). Interestingly, the first assay showed a higher correlation with the second assay (0.64) compared with the third assay (0.35) (**Table 1**). The first and second assays were conducted in two fall seasons, but the third assay was done in summer. In addition, the BLUP data of TGC2 showed a mean of 3.50 and correlation coefficients of 0.74 (vs. the third assay) to 0.84 (vs. the second assay) (**Table 1**). For the combined population, additional BLUP data were generated with six phenotypic data sets of both TGC1 and TGC2 and showed normal distributions with a mean of 3.09 (**Figure 1C**). The BLUP data of the three populations (TGC1, TGC2, and combined) were used to assess the prediction accuracy of GEBVs for BW resistance.

**Table 1:** Correlations between three independent seedling assays and best linear unbiased prediction (BLUP) for bacterial wilt resistance in two tomato germplasm collections: TGC1 and TGC2.

Collection	Phenotype	Pearson correlation coefficient		
		2nd assay	3rd assay	BLUP
TGC1 (*n* = 162)	1^st^ assay	0.16	0.69	0.76
	2^nd^ assay	–	0.36	0.70
	3rd assay	–	–	0.87
TGC2 (*n* = 191)	1^st^ assay	0.64	0.35	0.83
	2^nd^ assay	–	0.42	0.84
	3^rd^ assay	–	–	0.74

### 3.2 Prediction accuracy of GEBVs between GS models

The 51K SNP array generated common 31,142 SNPs with reliable polymorphisms in both tomato collections. Their genotypic data were used to train six GS models along with the BLUP data in three training populations (TGC1, TGC2, and combined). The parametric models, RR-BLUP, BayesA, and Bayesian LASSO, showed higher prediction accuracies in TGC2 (0.672–0.680) relative to the TGC1 (0.518–0.544) and combined (0.609–0.625) populations (**Table 2**). Of these models, Bayesian LASSO showed the highest prediction accuracy in both TGC1 (0.544) and combined (0.625) populations. The prediction accuracy of Bayesian LASSO in TGC2 was 0.672, which was slightly lower than 0.680 for RR-BLUP. Similarly, three non-parametric models (RKHS, SVM, and random forest) revealed higher prediction accuracies in TGC2 (0.595–0.683) relative to the TGC1 (0.451–0.526) and combined (0.549–0.614) populations (**Table 2**). In addition, random forest provided the highest prediction accuracy in both TGC2 (0.683) and combined (0.614) populations, while SVM resulted in the lowest prediction accuracy in all the populations. With these results, Bayesian LASSO and random forest were selected as the best models to predict GEBVs for BW resistance and used to identify low-density SNP sets for genomic selection.

**Table 2:** The prediction accuracy of six genomic selection models for bacterial wilt resistance in the three training populations (TGC1, TGC2, and combined) based on 31,142 SNP markers.

Genomic selection model		Prediction accuracy^a^		
		TGC1 (*n* = 162)	TGC2 (*n* = 191)	Combined (*n* = 353)
Parametric models	RR-BLUP	0.537	0.680	0.621
	BayesA	0.518	0.672	0.609
	Bayesian LASSO	0.544	0.672	0.625
Non-parametric models	RKHS	0.526	0.669	0.611
	SVM	0.451	0.595	0.549
	Random forest	0.506	0.683	0.614

RR-BLUP, ridge regression best linear unbiased prediction; RKHS, reproducing kernel Hilbert space; SVM, support vector machine.

a Prediction accuracy was determined using the Pearson correlation coefficients between genomic-estimated breeding values (GEBVs) and BLUP. GEBVs were estimated using the leave-one-out cross-validation method in each model.

### 3.3 Efficiency of low-density SNP markers for genomic selection

**Figure 2:**
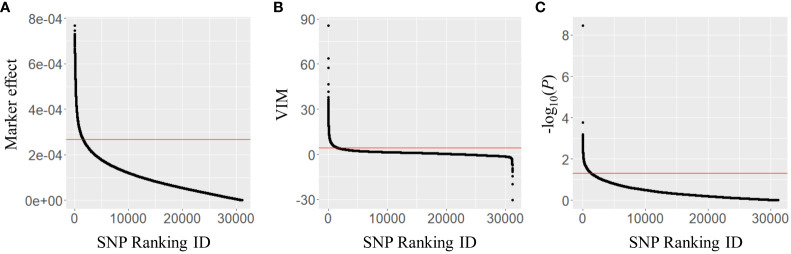
Distribution of **(A)** marker effects, **(B)** variable importance (VIM) values, and **(C)** −log_10_(*P*-values) of 31,142 SNP markers obtained in the combined population (*n* = 353). The marker effects and VIM values were obtained using Bayesian LASSO and random forest, respectively. The *P*-values were derived from the multilocus mixed model (MLMM) in a genome-wide association study. The red lines represent the thresholds for marker selection, with the top 5% of marker effects, VIM values, and *P*-value of 0.05.

The marker effects of 31,142 SNPs were estimated using Bayesian LASSO in the combined population and 1,557 SNPs in the top 5% were selected to generate a subset of markers (**Figure 2A**). Similarly, the second subset of 1,557 SNPs was produced using the VIM values, which were calculated in random forest (**Figure 2B**). For the third subset, GWAS for BW resistance was conducted in the combined population. Population structure analysis revealed that 353 tomato accessions were separated into seven clusters, and the number of accessions per cluster ranged from 10 (cluster 7) to 102 (cluster 6) (**Supplementary Table S1**). The majority of accessions in clusters 1 (88.0%) and 2 (89.7%) were derived from TGC1, while clusters 6 and 7 were represented by the TGC2 accessions (89.2% and 100.0%). In the other clusters, we found relatively high levels of mixture (31.9%–68.1% for each collection). The multilocus mixed model (MLMM) identified 63 SNPs significantly associated with BW resistance at *P <*0.005 on nine chromosomes (1, 2, 4, 6–9, 11, 12) including three major QTL (*Bwr-4*, *Bwr-6*, and *Bwr-12*) (**Supplementary Figure S1**, **Supplementary Table S2**). With the threshold of *P <*0.05, a total of 1,357 SNPs showed significant associations across 12 chromosomes (**Figure 2C**). This number of SNPs could be large enough to capture QTL with minor effects and, thus, was used to generate the third subset.

**Figure 3:**
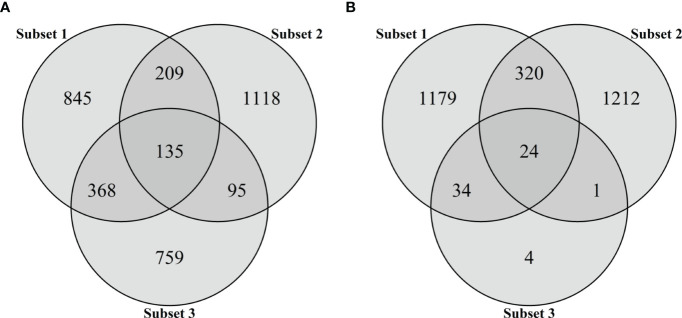
Venn diagram for the number of SNPs in three subsets based on marker effects (subset 1), variable importance values (subset 2), and marker–trait associations (subset 3). The intersections of circles represent the number of common SNPs of two or three subsets. Subsets 1 and 2 consist of 1,557 SNPs representing the top 5% of marker effects or VIM values, respectively. The SNP markers significantly associated with BW resistance were selected as subset 3 using two thresholds: **(A)** *P* < 0.05 and **(B)** *P* < 0.005.

Each subset included not only subset-specific but also common SNPs, which were found in more than two subsets (**Figure 3**). For the first subset, 845 (54.3%) of 1,557 SNPs were subset-specific and other SNPs were also present in the second (344 SNPs) and third (503 SNPs) subsets. Of the 1,557 SNPs, 1,118 (71.8%) in the second subset were subset-specific, while 230 SNPs were also found in the third subset. Moreover, a total of 135 SNPs were found in all the subsets (**Figure 3A**). With only 63 significant SNPs at *P <*0.005 in the third subset, we found much smaller numbers of common SNPs in all pairwise comparisons, ranging from 24 to 344 (**Figure 3B**). Therefore, the significant 1,357 SNPs at *P <*0.05 were used as the third subset to generate two additional subsets. The fourth subset consisted of 3,529 that were obtained by combining all the SNPs in the first to third subsets, while the fifth subset included 135 common SNPs (**Figure 3A**).

**Figure 4:**
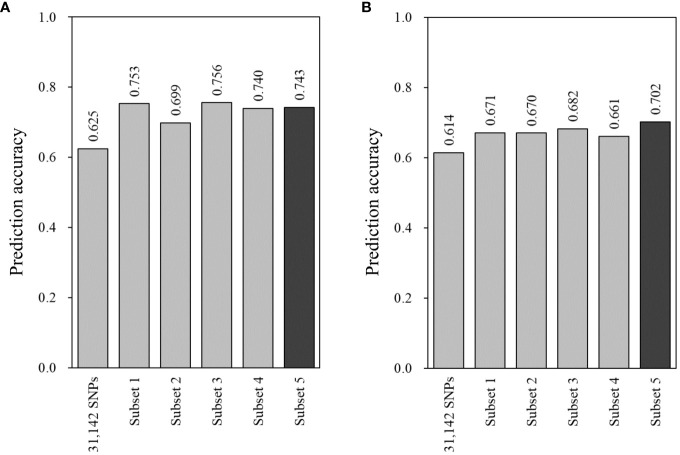
Prediction accuracy of genomic estimated breeding values (GEBVs) with the 31,142 SNPs and five subsets for bacterial wilt resistance in the combined population (*n* = 353). GEBVs were estimated using two models: **(A)** Bayesian LASSO and **(B)** random forest with leave-one-out cross-validation. Prediction accuracy was determined with the Pearson correlation coefficients between GEBVs and the observed phenotypes. Three subsets were generated based on marker effects (subset 1, *n* = 1,557), variable importance values (subset 2, *n* = 1,557), and marker–trait associations (subset 3, *n* = 1,357). All the SNPs of these subsets were combined for subset 4 (*n* = 3,529), and the common SNPs of the three subsets were used for subset 5 (*n* = 135).

Five SNP subsets were assessed for prediction accuracy using two GS models, Bayesian LASSO and random forest, in the combined population. With Bayesian LASSO, all the subsets showed higher prediction accuracies compared with 0.625 in the total set of 31,142 SNPs (**Figure 4**). Of these, the first (marker effect-based) and third (GWAS-based) subsets resulted in 0.753 and 0.756 that were higher than 0.699 of the second subset (VIM-based). Interestingly, the fourth and fifth subsets, which consisted of 3,529 and 135 SNPs, showed a little difference in prediction accuracy (0.740 vs. 0.743) (**Figure 4**). The random forest model also provided higher prediction accuracies in the five subsets (0.661 to 0.702) compared with the total set (0.614). However, the first to fourth subsets showed similar levels of prediction accuracy ranging from 0.661 to 0.682. In addition, the highest prediction accuracy was found in the fifth subset (**Figure 4**).

## 4 Discussion

GS was proposed to increase genetic gains for quantitative traits by predicting GEBVs with genome-wide molecular markers (Meuwissen et al., 2001). In the present study, we investigated GS for BW resistance using two tomato collections, TGC1 and TGC2. Different phenotypic distributions and correlations were observed between three seedling assays for disease evaluation in both collections, suggesting that environmental effects were present. For BW, high temperatures (30°C–35°C) are known to increase susceptibility in tomato (Lee et al., 2011; Singh et al., 2014; Yeon et al., 2022). Therefore, the phenotypic data of each collection were adjusted using BLUP, which accounts for random effects (Henderson, 1975; Robinson, 1991). Furthermore, the TGC1 and TGC2 data were integrated based on BLUP to produce a large training population. The size and genetic diversity of the training populations are factors that affect the prediction accuracy of GEBVs (Desta and Ortiz, 2014; Edwards et al., 2019). The resulting BLUP data in the three training populations (TGC1, TGC2, and combined) were used to evaluate the prediction accuracies of GEBVs between different GS models and develop a cost-effective strategy for BW resistance.

Several GS models have been developed to estimate GEBVs for traits of interest based on different assumptions (Zhong et al., 2009; Daetwyler et al., 2010; Desta and Ortiz, 2014). Parametric models are commonly used to estimate additive genetic effects, while non-parametric models are appropriate for non-additive genetic effects and multivariates (De Los Campos et al., 2010; Holliday et al., 2012; Pérez-Rodríguez et al., 2012; Krishnappa et al., 2021). In this study, the GEBVs of BW resistance were estimated using both parametric (RR-BLUP, BayesA, and Bayesian LASSO) and non-parametric (RKHS, SVM, and random forest) models. Of these, Bayesian LASSO and RR-BLUP showed better performances than the non-parametric models, except random forest in TGC2. For BW resistance, additive genetic effects with lack of epistasis were previously reported in tomato (Da Silva Costa et al., 2018; Costa et al., 2019). In addition, three major QTL (*Bwr-4*, *Bwr-6*, and *Bwr-12*) have been known to be associated with BW resistance along with several minor QTL (Thoquet et al., 1996a, Thoquet et al., 1996b; Wang et al., 2013; Nguyen et al., 2021). This genetic control of BW resistance supports that the parametric models are more appropriate to predict GEBVs than the non-parametric models. Since the Bayesian methods have various degrees of shrinkage for marker effects due to their prior distributions (De Los Campos et al., 2009; Wang et al., 2018), we found different prediction accuracies between BayesA and Bayesian LASSO. For RR-BLUP, all markers are assumed to have equal variances with small effects, and this model is known to be appropriate for complex traits controlled with several minor QTL (Meuwissen et al., 2001; Wang et al., 2018). Similar levels of prediction accuracy between Bayesian LASSO and RR-BLUP were also found for fruit traits in hot pepper (Hong et al., 2020). These results demonstrate that model performance depends on the complexity of quantitative traits.

Marker density is an important factor that affects the prediction accuracy of GEBVs for traits of interests (Heffner et al., 2009; Heffner et al., 2011a, Heffner et al., 2011b; Desta and Ortiz, 2014; Crossa et al., 2017). High-density markers across all the chromosomes have been commonly used to preserve linkage disequilibrium between markers and QTL for GS. However, a simulation-based study proposed that using markers associated with major QTL as fixed effects increases the prediction accuracy in RR-BLUP (Bernardo, 2014). This strategy has been utilized to estimate accurate GEBVs for agronomic traits and disease resistance in wheat (Bentley et al., 2014; Rutkoski et al., 2014; Sarinelli et al., 2019), pro-vitamin A content in maize (Owens et al., 2014), agronomic traits in rice (Spindel et al., 2016), and capsaicinoid content in hot pepper (Kim et al., 2022). In the present study, we also used marker–trait associations to identify low-density markers with high prediction accuracy. A total of 1,357 SNPs were selected based on significant associations with BW resistance at *P <*0.05. This GWAS-based subset included markers for all the known QTL with large effects. Furthermore, the 31,142 SNPs were filtered based on the top 5% of marker effects and VIMs to generate two subsets consisting of 1,557 SNPs. Two additional subsets were also produced using all the markers (3,529 SNPs) and common markers (135 SNPs) of the three subsets. All the subsets revealed higher levels of prediction accuracy than the 31,142 SNPs in the selected parametric (Bayesian LASSO) and non-parametric (random forest) models. As expected, Bayesian LASSO showed better performance in these subsets compared with random forest. Moreover, the marker effect-based and GWAS-based subsets resulted in higher prediction accuracies (0.753 and 0.756) relative to the VIM-based subset (0.699) in Bayesian LASSO. We also found that the subset of 135 SNPs showed slightly higher prediction accuracy (0.743) relative to the subset of 3,529 SNPs (0.740). These results demonstrate that a cost-effective marker set for GS can be developed based on marker effects and marker–trait associations. The VIM values are also suggested to be useful to filter markers, depending on the genetic architecture of traits.

In conclusion, our study was conducted to investigate a cost-effective GS approach with low-density markers for improving BW resistance in cultivated tomato. The prediction accuracy of GEBVs was evaluated in six GS models representing parametric (RR-BLUP, BayesA, and Bayesian LASSO) and non-parametric (RKHS, SVM, and random forest) models. Of these, Bayesian LASSO and RR-BLUP resulted in higher prediction accuracies relative to the non-parametric models in two of three training populations. In addition, random forest showed better performance than the other non-parametric models. The 31,142 SNPs were filtered to generate five subsets using marker effects, variable importance values, and marker–trait associations. All the subsets with low-density were effective to estimate more accurate GEBVs compared with the high-density marker set. Moreover, we found a high level of prediction accuracy in the subset of 135 SNPs, which were selected as common markers among the three subsets based on marker effects, variable importance values, and marker–trait associations. These results suggest that low-density markers can be effective to predict accurate GEBVs, depending on the complexity of quantitative traits. The SNP subsets from the present study will be valuable to accelerate the practical application of GS for improving BW resistance in tomato breeding programs.

## Data availability statement

The genotypic data of tomato collections are available in the Mendeley Data repository, https://data.mendeley.com/datasets/bxcpc274fh/1.

## Author contributions

JY: Data curation, Formal analysis, Investigation, Methodology, Writing – original draft. NL: Data curation, Investigation, Methodology, Writing – original draft. JH: Data curation, Formal analysis, Writing – review & editing. SS: Conceptualization, Funding acquisition, Investigation, Methodology, Supervision, Writing – review & editing.
