# Supplementary material for: Low-density SNP markers with high prediction accuracy of genomic selection for bacterial wilt resistance in tomato
Source: Front Plant Sci. 2024 May 30;15:1402693. doi: 10.3389/fpls.2024.1402693 (PMC11169939; doi:10.3389/fpls.2024.1402693)
Supplement: Supplementary file 1 [file DataSheet_1.pdf]

Supplementary Material

Low-density SNP markers with high prediction accuracy of genomic selection to improve bacterial wilt resistance in tomato

Running title: Core sets of SNPs for genomic selection of bacterial wilt resistance in tomato

Jeyun Yeon[1], Ngoc Thi Le[1], Jaehun Heo[1], and Sung-Chur Sim[1,2*]

*** Correspondence:** Sung-Chur Sim: sungchur@sejong.ac.kr

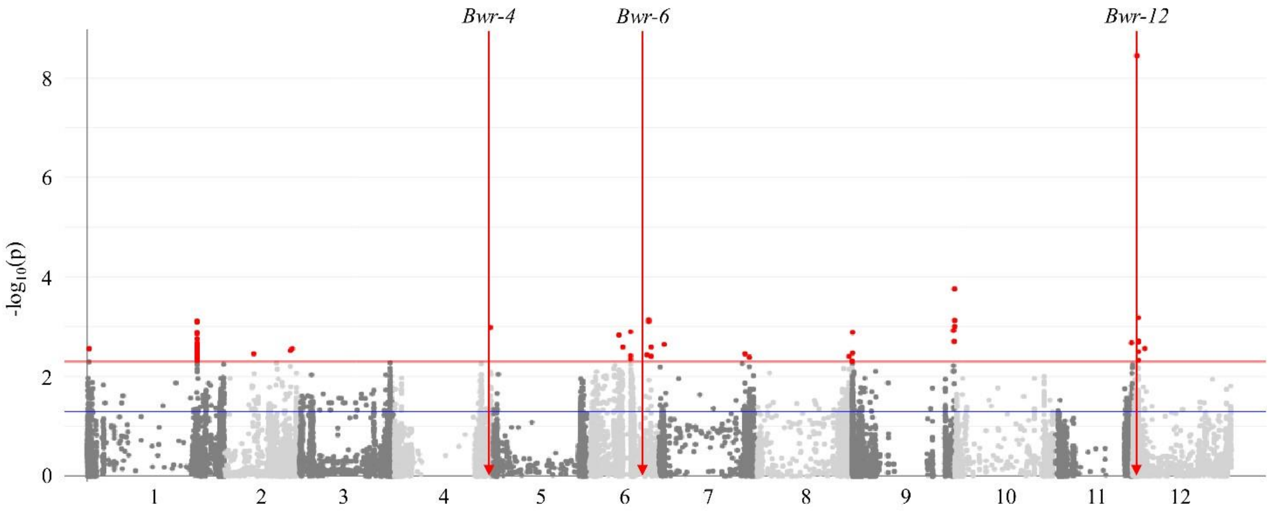

Figure S1. Manhattan plot of a genome-wide association study for bacterial wilt resistance in the combined population (n=353). The horizontal lines indicate two thresholds of $P < 0.005$ (red) and $P < 0.05$, (blue). A total of 31,142 genome-wide SNPs over 12 chromosomes are shown with gray dots and red dots represent SNPs significantly associated with bacterial wilt resistance at $P < 0.005$. The red arrows indicate the positions of SNPs associated with the major QTL, *Bwr-4*, *Bwr-6*, and *Bwr-12* (Nguyen et al., 2021).
